# Functional and topographic effects on DNA methylation in *IDH1/2* mutant cancers

**DOI:** 10.1038/s41598-019-53262-7

**Published:** 2019-11-14

**Authors:** Ramona Bledea, Varshini Vasudevaraja, Seema Patel, James Stafford, Jonathan Serrano, Gianna Esposito, Lilian M. Tredwin, Nina Goodman, Andreas Kloetgen, John G. Golfinos, David Zagzag, Britta Weigelt, A. John Iafrate, Erik P. Sulman, Andrew S. Chi, Snjezana Dogan, Jorge S. Reis-Filho, Sarah Chiang, Dimitris Placantonakis, Aristotelis Tsirigos, Matija Snuderl

**Affiliations:** 1 Department of Pathology, NYU Langone Health and School of Medicine, New York, NY USA; 2 Department of Neurological Sciences, University of Vermont, Larner College of Medicine, Burlington, VT USA; 3 Department of Neurosurgery, NYU Langone Health and School of Medicine, New York, NY USA; 4 Department of Pathology, Memorial Sloan Kettering Cancer Center, New York, NY USA; 5 Department of Pathology, Massachusetts General Hospital, Boston, MA USA; 6 Department of Radiation Oncology, NYU Langone Health and School of Medicine, New York, NY USA; 7 Laura and Isaac Perlmutter Cancer Center, NYU Langone Health and School of Medicine, New York, NY USA; 8 Kimmel Center for Stem Cell Biology, NYU Langone Health and School of Medicine, New York, USA; 9 Neuroscience Institute, NYU Langone Health and School of Medicine, New York, USA

**Keywords:** Methylation analysis, Cancer genomics, Microarrays, Molecular medicine

## Abstract

*IDH1/2* mutations are early drivers present in diverse human cancer types arising in various tissue sites. *IDH1/2* mutation is known to induce a global hypermethylator phenotype. However, the effects on DNA methylation across IDH mutant cancers and functionally different genome regions, remain unknown. We analyzed DNA methylation data from *IDH1/2* mutant acute myeloid leukemia, oligodendroglioma, astrocytoma, solid papillary breast carcinoma with reverse polarity, sinonasal undifferentiated carcinoma and cholangiocarcinoma, which clustered by their embryonal origin. Hypermethylated common probes affect predominantly gene bodies while promoters in *IDH1/2* mutant cancers remain unmethylated. Enhancers showed global hypermethylation, however commonly hypomethylated enhancers were associated with tissue differentiation and cell fate determination. We demonstrate that some chromosomes, chromosomal arms and chromosomal regions are more affected by *IDH1/2* mutations while others remain resistant to *IDH1/2* mutation induced methylation changes. Therefore *IDH1/2* mutations have different methylation effect on different parts of the genome, which may be regulated by different mechanisms.

## Introduction

The Isocitrate dehydrogenase (*IDH*) gene family is composed of three genes, *IDH1*, *IDH2* and *IDH3*. IDH enzymes catalyze oxidative decarboxylation of isocitrate to alpha-ketoglutarate (α-KG) in the citric acid cycle. While *IDH1* is located in the cytosol and peroxisome, *IDH2/3* are located in mitochondria. Mutations in the *IDH1* and *IDH2* genes have been found in various tumor types and catalyze reduction of a-KG into a structurally similar oncometabolite 2-hydroxyglutarate (2-HG), which probably functions as an α-KG antagonist in numerous metabolic processes^1,2^. 2-HG is normally produced at a low level (<300 µM) by errors in catalysis during phosphoglycerate dehydrogenase, hydroxyl oxoacetic transferase and malate dehydrogenase reactions. It has no known metabolic function in mammals, and is rapidly cleared via conversion to αKG by chirality-specific dehydrogenases (D-2HGDH or L-2HGDH) in physiologic conditions. In cancers with *IDH1/2* mutations, 2-HG levels can reach millimolar concentrations saturating normal clearing mechanisms.

Among other effects, 2-HG accumulation inhibits histone demethylases and TET family 5-methylcytosine hydroxylases. Mutations in *IDH1/2* genes have been identified in acute myeloid leukemia^3–6^, glioma^7–9^, cholangiocarcinoma^10,11^, sinonasal undifferentiated carcinoma^12,13^, chondrosarcoma and periosteal chondroma^14,15^, solid papillary carcinoma with reverse polarity, a rare morphologic subtype of breast carcinoma^16^, and also occur rarely in other tumors like medulloblastoma^17^. All somatic *IDH* mutations occur in key residues within the active site, with hotspots in one of three arginine residues critical for isocitrate binding^18^, specifically *IDH1* R132 and the *IDH2* R172 and R140 codons. Regardless of tumor type, *IDH* mutant cancers show global DNA hypermethylation when compared to their wild-type counterparts. One effect of DNA hypermethylation is blockade of cell differentiation, which can be reversed by targeted inhibition^3,19–22^. In leukemias and gliomas, *IDH* mutations occur early in tumorigenesis^5,8,9,23^ and appear insufficient in driving tumor growth alone unless paired with *ATRX* loss^24,25^ and *TP53* mutation^25^, or loss of 1p/19q.

The prognostic value of *IDH1/2* mutations seems to be disease dependent. While in gliomas, *IDH1/2* mutations are associated with favorable outcome^7,26^, in other solid tumors and leukemia^27–30^ the effect on prognosis is either less favorable or unclear^31,32^. In addition, *IDH1/2* targeted therapy has variable responses among *IDH* mutant cancer types. Since the biochemical effect of *IDH* mutations is presumably the same in all cancer and cell types, this suggests that the *IDH* mutations may have different effect in different cell types.

In this study, we first analyzed tumor types for which biologically relevant wild-type tumors are available for comparisons. In addition, we also compared DNA methylation profiles of *IDH1/2* mutated AML to normal blood controls. Next, we sought to elucidate the effects of *IDH1/2* mutations on DNA methylation across six *IDH* mutant cancer types without wild-type tumor comparisons by identifying common hyper- and hypomethylated probes shared among all *IDH1/2* mutant tumor types. While *IDH1/2* mutations have been associated with the CpG island hypermethylation, we sought to elucidate whether functionally different parts of the genome, such as gene body, promoter or enhancers are differentially affected by *IDH* mutation induced DNA methylation changes. We evaluated whether entire or specific parts of chromosomes show different effect of *IDH* mutation induced hypermethylation. We hypothesized that DNA hyper- and hypomethylation changes that are common across all *IDH1/2* mutated cancers of various tissues of origin may represent universal effect of the *IDH1/2* mutations on the DNA methylation. Lastly, we analyzed the effect of common hyper- and hypomethylated gene bodies, enhances and promoters on biologic pathways to identify biologic processes shared among all *IDH1/2* mutated cancers in our study.

## Results

### Tumor characteristics

In this study we incorporated DNA methylation data from Cancer Genome Atlas (TCGA) profiled IDH mutant and wild-type acute myeloid leukemia (AML, N = 21)^6^ and cholangiocarcinoma (N = 9)^11^. In addition, a cohort of normal non-neoplastic blood samples of patients evaluated for leukocytosis (n = 32) was used for comparisons with IDH mutant AML. DNA methylation data from our previously published studies on solid papillary breast carcinoma with reverse polarity (N = 5)^16^, and sinonasal undifferentiated carcinoma (SNUC, N = 8)^12^, as well as DNA methylation profiles of *IDH* mutant oligodendroglioma (N = 20) and astrocytoma (N = 31) profiled at NYU Langone Health from 2015 to 2018. IDH1/2 mutant brain tumors were compared with IDH wild-type glioblastoma from our previously published cohort^33^. A cohort of proneural (RTKI, n = 10) GBMs was used for comparison with the oligodendroglioma cohort and mix of classic and mesenchymal GBMs (n = 10 for each subtype) was used for comparison with IDH mutant astrocytoma.

### *IDH1/2* mutation induced hypermethylation is prominent in gene bodies and enhancers while promoters show global hypomethylation

**Figure 1:**
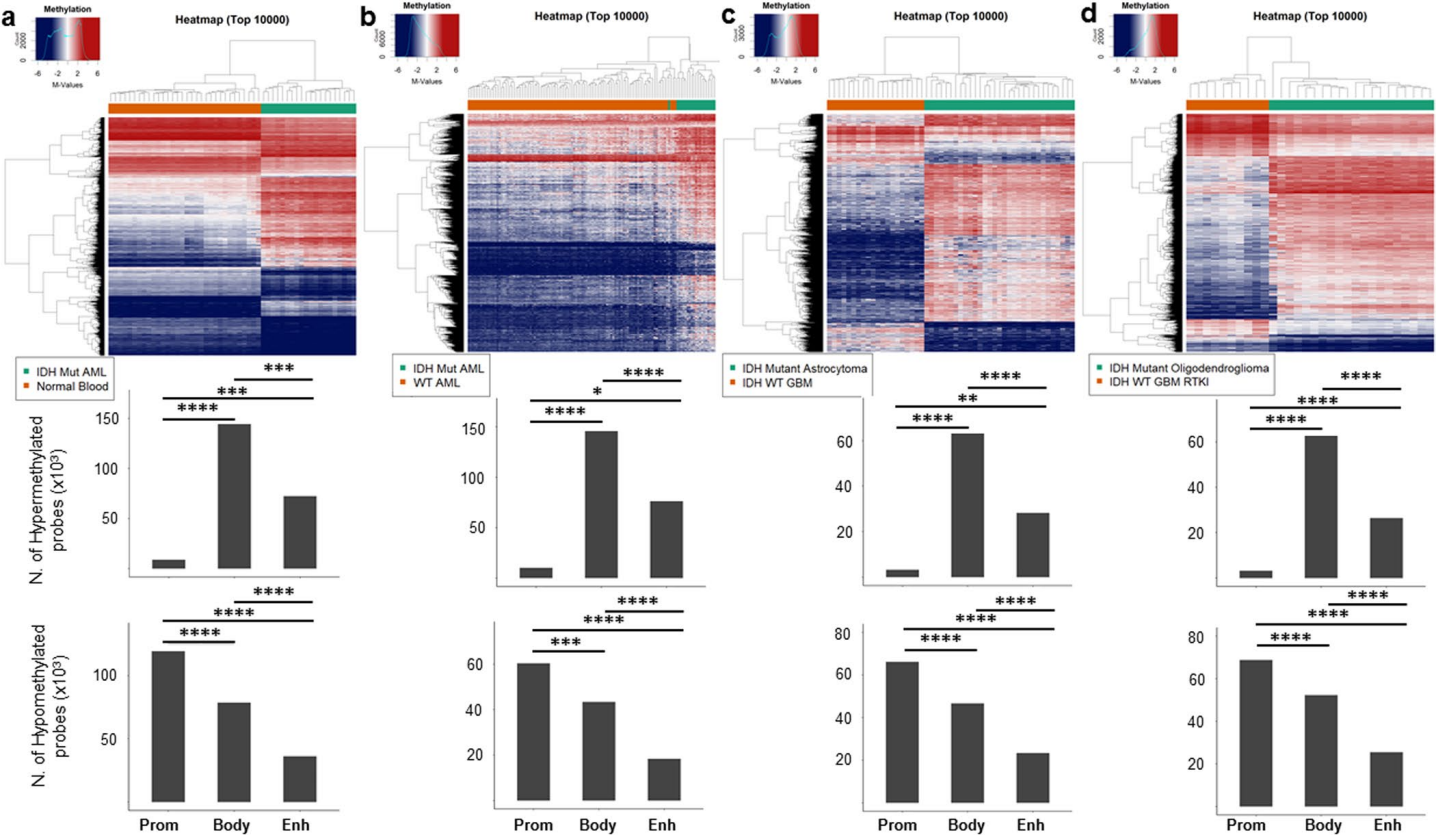
Disease specific distribution of hyper- and hypomethylated probes among functional regions. Differential methylation analysis of *IDH1/2* mutated AML compared to normal blood (**a**) and IDH wild-type AML (**b**), *IDH1/2* mutated astrocytoma compared to IDH wild-type GBM (Classic and Mesenchymal subtypes) (**c**) and IDH mutated oligodendroglioma compared to RTKI (Proneural) GBM (**d**). Heatmaps show clustering using top 10,000 most differentially methylated probes. Bar plots represent the number of all significant (FDR < 0.05) hyper- and hypomethylated probes (x10^3^) in each IDH mutated tumor when compared to the wild-type counterpart. Gene bodies and enhancers show significantly higher number of hypermethylated probes than promoters while promoters show significantly higher number of hypomethylated probes, across all comparisons. Two-tailed t-test, p-value: *<1 × 10^−2^, **<1 × 10^−3^, ***<1 × 10^−4^ and ****<1 × 10^−5^.

We compared hyper- and hypomethylation of *IDH1/2* mutated tumors with biologically relevant IDH wild-type tumors when available. DNA methylation of IDH mutated AML was compared both with normal non-neoplastic blood DNA sample (leukocytes) and IDH wild-type AML (Fig. 1a,b, and Supp. Fig. 1a,b, respectively). *IDH1/2* mutated astrocytoma was compared to a balanced cohort of Classic (RTKII) and Mesenchymal glioblastoma (GBM) (Fig. 1c and and Supp. Fig. 1c) and oligodendroglioma was compared to a cohort of Proneural (RTKI) GBM (Fig. 1d and and Supp. Fig. 1d). The analysis of IDH mutated and wild-type cholangiocarcinoma showed a very low number of probes with FDR < 0.05 (n = 980) and was excluded from further analysis. Differential methylation analysis showed that in all four tumor specific comparisons, *IDH1/2* mutated tumors had a significantly higher number of hypermethylated gene body and enhancer probes than promoters and significantly higher number of hypomethylated promoter probes than gene bodies and enhancers (Fig. 1, Supp. Fig. 1). The disease specific analysis showed that there was a significant variability in the number of hyper- and hypomethylated probes, with IDH mutated AML showing the highest number of hypermethylated probes (Fig. 1a,b) compared to IDH mutant Astrocytoma (Fig. 1c) and Oligodendroglioma (Fig. 1d), suggesting that other factors such as concurrent driver mutations of IDH wild-type tumors or the tissue of origin play role in tumor type specific methylation changes. Interestingly, IDH mutated AML compared to normal blood showed the highest number of differentially hypomethylated promoter probes compared to IDH mutated vs wild-type AML, oligodendroglioma vs RTKI GBM and astrocytoma vs Classic/Mesenchymal (Fig. 1, Supp. Fig. 1) comparisons. Overall, these findings suggest that global hypermethylation induced by *IDH1/2* mutations affects the genome in a function specific manner with strong propensity for hypermethylation of enhancers and gene bodies, while promoters remain hypomethylated.

### *IDH1/2* mutation induced methylation changes across different tumor tissues

**Figure 2:**
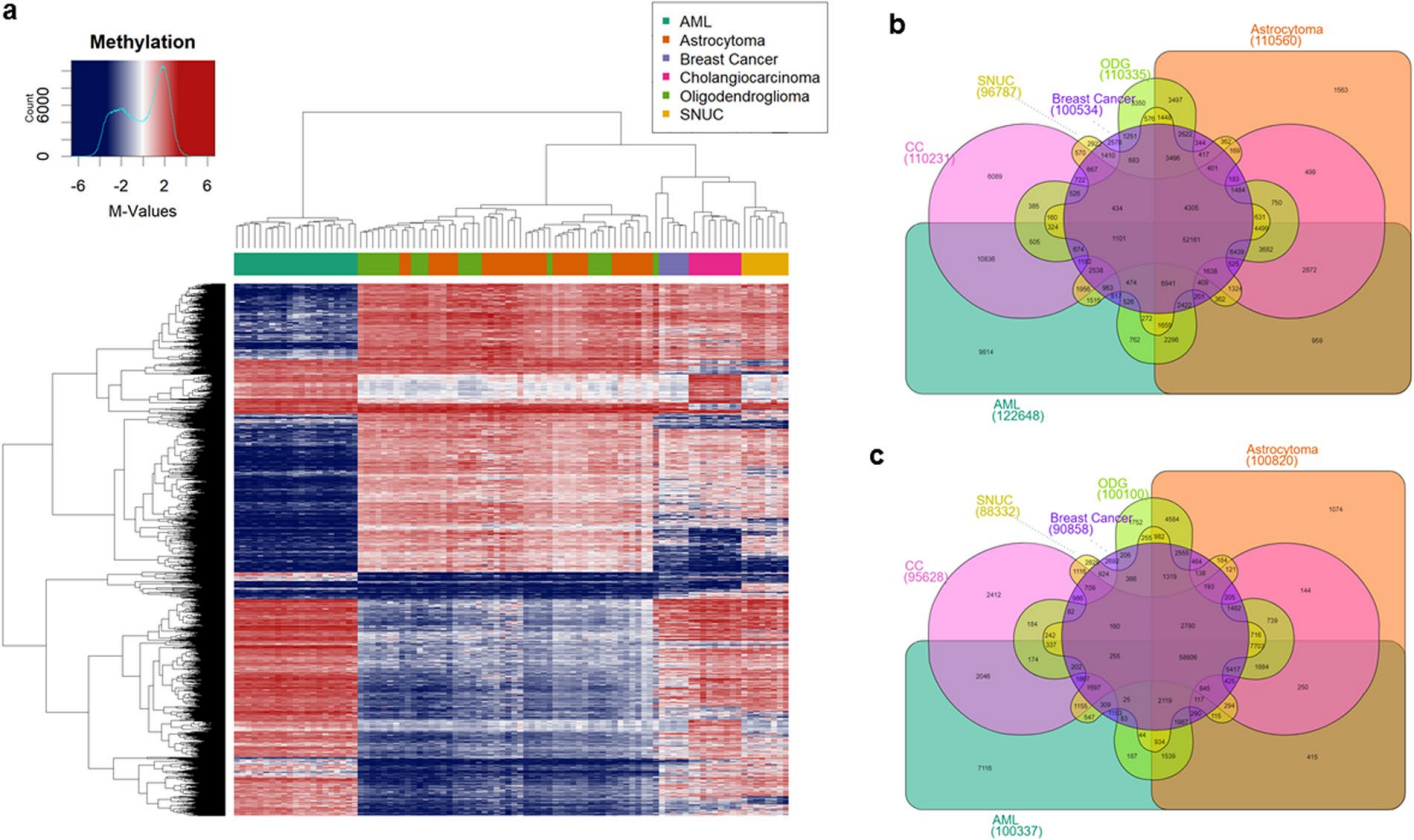
DNA Methylation across six *IDH1/2* mutant cancers. (**a**), Supervised hierarchical clustering of acute myeloid leukemia (AML), astrocytoma, breast cancer, cholangiocarcinoma (CC), oligodendroglioma (ODG), and sinonasal undifferentiated carcinoma (SNUC) using top 10,000 differentially methylated probes shows that despite all tumors harboring *IDH1* or *IDH2* mutations, tumors cluster along the developmental lines with mesenchymal (AML), neuroectodermal (astrocytoma, oligodendroglioma) and epithelial (CC, SNUC and breast carcinoma) cancers. (**b**,**c)**, Venn diagram shows shared and unique hypermethylated (**b**) and hypomethylated (**c**) probes for each disease. AML has the highest number of total and unique hyper- and hypomethylated probes.

Mutations in *IDH1* and *IDH2* genes lead to global hypermethylation which readily distinguishes mutated tumors from their wild-type counterparts^3,34^ (Fig. 1). However, what DNA methylation changes are common across different *IDH1/2* mutated cancers remains unclear. We performed differential methylation analysis of *IDH1/2* mutant tumors comparing six different tumor types. We observed that tumors formed three distinct clusters corresponding to the embryonal origin of the tissue (Fig. 2a, top 10,000 differentially methylated probes shown). The first group included acute myeloid leukemia (AML), which is derived from mesoderm. The second cluster was formed by neuroectoderm-derived tumors oligodendroglioma and astrocytoma and the third cluster included breast carcinoma, sinonasal undifferentiated carcinoma (SNUC) and cholangiocarcinoma, which are derived from epithelial tissues. However, SNUC and cholangiocarcinoma, which are both derived from the endodermal layer, formed a subgroup while breast carcinoma samples, which are derived from the ectoderm formed its own subgroup, albeit a single oligodendroglioma, which is derived from the neuroectoderm, clustered with breast carcinomas. Despite *IDH1/2* mutations supposedly affecting the same enzymes in all cancers, all tumors still clustered based on their tissue of origin and more specifically the embryonal layer. Since *IDH1/2* mutations are early drivers^4,9,23,25^ this suggests that *IDH1/2* mutation induced DNA methylation changes act on the developmental background and *IDH1/2* induced DNA methylation remains tissue of origin specific.

Since not all cancers in our study have biologically relevant wild-type counterparts available for comparison, we sought to identify common hyper- and hypomethylated probes in our *IDH1/2* mutated tumors by identifying hyper- and hypomethylated probes, which are common across all six IDH1/2 mutated tumor types in our study. When analyzing methylated probes across six tumor types, we observed that only a fraction of probes was hyper- or hypomethylated only in one tumor type, while large portion of the probes are hyper- and hypomethylated in two or more cancer types (Fig. 2b,c, Table 1). Approximately 1.4% (1,563/110,560) and 8% (9,814/122,648) probes were hypermethylated only in *IDH* mutant astrocytomas and acute myeloid leukemias, respectively. In total, 52,161 probes were hypermethylated in all tumor types analyzed in our study. However, while the number of hypermethylated probes was ~100,000 in most of the cancers, acute myeloid leukemia (AML) showed the most prominent hypermethylation phenotype with 122,648 hypermethylated probes and the highest level of tumor specific hypermethylation (Fig. 2b, Table 1), validating the findings observed in *IDH1/2* mutant vs wild-type leukemia analysis (Fig. 1a,b). Astrocytomas showed a number of hypermethylated probes that was similar to oligodendroglioma and cholangiocarcinoma, but higher than SNUC and breast carcinoma. However, the fraction of tumor type specific hypermethylated probes was the lowest (1.4%) among astrocytomas, which may be due to a large number of probes which are overlapping with oligodendroglioma. SNUC showed the lowest number of hypermethylated probes. Similarly, the number of hypomethylated probes varied across tumors as well (Fig. 2c, Table 1). When analyzing the hypomethylated probes, we observed that their total number was lower across all cancer types compared to the hypermethylated probes, with SNUC having the lowest number of hypomethylated probes and astrocytoma again showing the lowest number of tumor specific hypomethylated sites (1.1%).

**Table 1:** Number of hyper- and hypomethylated probes is IDH mutant cancers.

Tumor Type	N. of hypermethylated probes	Tumor specific hypermethylated probes	% of tumor specific
AML	122648	9814	8.0
Astrocytoma	110560	1563	1.4
Oligodendroglioma	110335	3350	3.0
Breast cancer	100534	2578	2.6
Cholangiocarcinoma	110231	6089	5.5
SNUC	96787	2922	3.0
**Average**	**108516**	**4386**	**3.9**
	**N. of hypomethylated probes**	**Tumor specific hypomethylated probes**	
AML	100337	7116	7.1
Astrocytoma	100820	1074	1.1
Oligodendroglioma	100100	1752	1.8
Breast cancer	90858	2692	3.0
Cholangiocarcinoma	95628	2412	2.5
SNUC	88332	2825	3.2
**Average**	**96013**	**2978.5**	**3.1**

**Figure 3:**
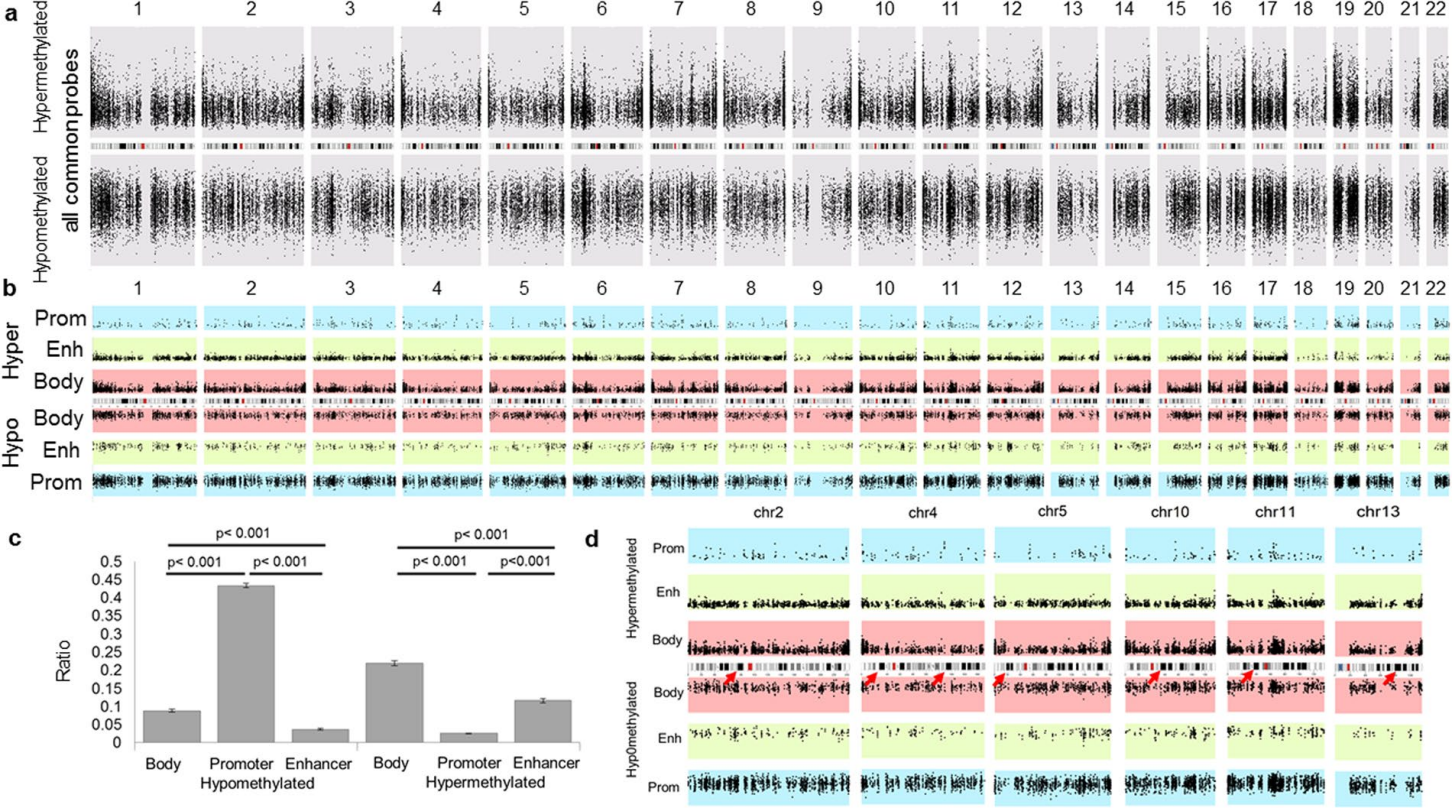
Genome wide distribution of common hyper- and hypomethylated probes. y axis scale ranges between 1.9 and 5.1 (**a**) Distribution of all common hyper- and hypomethylated probes across the genome (from Fig. 2b,c and Table 1). There was no significant difference when all hyper- and hypomethylated probes are plotted together. (**b**) Common hyper- (above the chromosomes) and hypo- (below the chromosomes) methylated probes in promoters (Prom, blue), Enhancers (Enh, green), and gene bodies (Body, red) are distributed across the human genome. For hypermethylated probes, the values range from +1.9 to +7 and for hypomethylated probes the range is between −1.9 and −7. Promoters show high density of hypomethylated probes, while gene bodies and enhancers show high density of hypermethylated probes across the human genome. Notably, enhancers show low density of hypomethylated probes. (**c**) The ratio of common hypomethylated promoter probes was significantly higher than common gene body and enhancers probes. In contrary, the ratio of common hypermethylated gene body probes is significantly higher than both enhancer and promoter probes. The ratio of common hypermethylated enhancer probes was also significantly higher than promoters. (paired t-test). (**d**) Closer look at selected chromosomes shows that specific regions in the human genome corresponding to the dark bands are largely devoid of hyper- and hypomethylated probes corresponding to gene body, enhancer and promoter (arrows).

Once we determined, which hyper- and hypomethylated probes are common across six *IDH1/2* mutant tumor types, we sought to determine the distribution of common hyper- and hypomethylated probes across the genome and elucidate what is the ratio of hyper- and hypomethylation in different regions of the genome. For this we plotted the probes across the genome and we calculated the ratio of common hyper-and hypomethylated probes per all probes in each chromosomal region. There was no difference among chromosomes when all probes were plotted (Fig. 3a). However in addition to the expected hypermethylation, we also observed a surprisingly high ratio of hypomethylation across the genome (Fig. 3a and Supp. Fig. 2) suggesting that *IDH1/2* mutation induced methylation changes are not limited to a hypermethylator phenotype and that certain regions and probes remain unmethylated in *IDH* mutant tumors.

**Figure 4:**
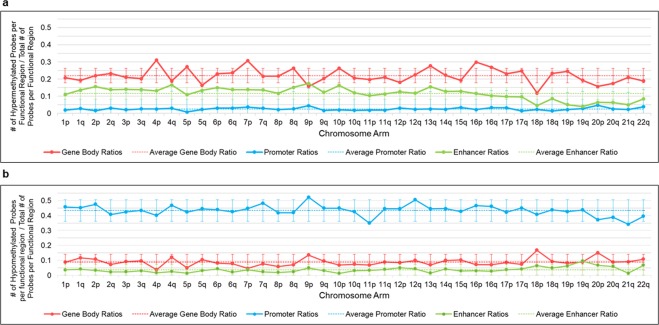
Quantification of the hyper- and hypomethylated ratios per chromosomal arms. To account for a variable coverage of probes for each functional region per chromosomal arm, we quantified the ratio of hyper/hypomethylated probes. (**a**) The ratio of common hypermethylated gene body, enhancer or promoter probes vs all gene body, enhancer or promoter probes, respectively in the particular chromosomal arm, shows highly variable effect of hypermethylation on promoters, enhancers and gene bodies, as well as variations between chromosomal arms. Notably, there is variation between different chromosomal arms even from the same chromosome (for example 4p vs 4q and 18p vs 18q). Promoters show low ratio of hypermethylated probes. (**b**) The ratio of common hypomethylated gene body, enhancer or promoter probes vs all gene body, enhancer or promoter probes, respectively in the particular chromosomal arm, shows high ratio of hypomethylated promoter probes compared to enhancers and gene body probes. Enhancers show the lowest ratio of hypomethylated probes. Short (p) arms of acrocentric chromosomes with no coverage on the Illumina arrays (13p, 14p, 15p, 21p, 22p) are not included in the analysis. Dotted lines represent averages of the ratios and error bars represent 95% CI.

CpG island hypermethylator phenotype is a hallmark of IDH mutant cancers^35^. However, as shown above by differential methylation of *IDH* mutant and wild-type tumors, gene bodies and enhancers showed hypermethylation while promoters show global hypomethylation. Therefore we aimed to confirm whether *IDH1/2* mutation induced hyper- and hypomethylation showed similar effect on promoters, enhancers and gene bodies across all six *IDH1/2* mutated cancer types including tumors in which we did not have the wild-type tumor or normal tissue for comparison. Therefore, we calculated ratios of common hyper-/hypomethylated probes in gene body/promoter/enhancer vs. all gene body/promoter/enhancer probes in each selected region. Similar to the *IDH1/2* mutant – IDH1*/2* wild-type analysis, the number of common hypermethylated promoter probes was relatively low across all chromosomes (Figs 3b,d, and 4a). In striking contrast, there was increased density of hypomethylated promoter probes across all chromosomes (Figs 3b,d, and 4b) with significantly higher ratio of hypomethylated promoter probes compared to ratios of hypomethylated enhancer (p-value < 0.001) and gene body (p-value < 0.001) probes (Fig. 3c). In contrast, gene bodies and enhancers showed significantly higher ratios of hypermethylated probes (Figs 3b,d and 4a) compared to ratio of hypermethylated promoters across all chromosomes (p-value < 0.001 and <0.001, respectively), (Fig. 3c).

### Chromosomes and specific chromosomal regions are differentially affected by *IDH1/2* mutation induced DNA methylation changes

In addition to the functional difference, we further explored whether there are topographic differences between chromosomes and chromosomal regions in addition to the functional variability in DNA methylation induced by *IDH1/2* mutations common in all six cancer types. We observed DNA methylation heterogeneity between chromosomes, chromosomal arms and even within specific chromosomal subregions/bands. We compared the ratios of commonly hypermethylated or hypomethylated vs all gene bodies, enhancers and promoters probes (Figs 3 and 4**)**. Gene bodies located on chromosomal arms 4p, 5p, 7p, 10p, 13q and chromosome 16 showed the highest fraction of hypermethylated probes, while chromosomal arms 5q, 9p, 18p and 20p showed the lowest ratio of hypermethylated gene body probes. Chromosomal arms 4q, 9p, 10p and 13q showed the highest ratio of hypermethylated enhancer probes across all *IDH1/2* mutant cancers. Considering that chromosomal arm 13p has virtually no methylation probe coverage on either 450k or EPIC array (Supp. Fig. 3), chromosome 13q enhancers and gene bodies appear the most affected by DNA hypermethylation across all *IDH1/2* mutant cancers. The second chromosomal arm with high ratios of both hypermethylated gene body and enhancer probes seems to be 10p, while 18p the lowest fraction of common gene body and enhancer hypermethylated probes in *IDH1/2* mutant tumors. When analyzing the ratios of hypomethylated probes, promoters showed relatively even levels across all chromosomes with 9p and 12q chromosomal arms showing the highest ratios of hypomethylated promoter probes and promoter probes on 11p and 21q showing the lowest fraction. There seemed to be a reverse relationship between the fractions of hyper and hypomethylated probes in gene bodies. Chromosomal arms 4p, 5p and 7p that showed high ratio of hypermethylated probes in gene bodies (Fig. 4a) tended to exhibit a particularly low fraction of the hypomethylated probes (Fig. 4b) while chromosomal arm 18p with high fraction of hypomethylated probes showed the lowest fraction of hypermethylated probes. Although enhancers showed a generally high ratio of hypermethylated probes and low fraction of hypomethylated probes, a similarly inverse relationship between hyper- and hypomethylated probes was not observed. The exception was 19q, which had the lowest fraction of hypermethylated enhancer probes and the highest fraction of hypomethylated enhancer probes. This pattern was also noted in 18p.

The analysis how gene bodies, enhancers and promoters are affected by hyper- and hypomethylation in tumor specific manner is limited by a highly variable number of cases, with some groups being extremely small due to the rarity of tumors. Furthermore, there is also significant underlying genetic heterogeneity in each of these diseases likely responsible for high variability between different tumor types (Figs 1, 2 and Supp. Fig. 1).

We also observed that certain chromosomal regions almost completely lacked any common hyper- or hypomethylated probes in enhancers, promoters or gene bodies, suggesting that these regions do not contain gene body/promoter or enhancer probes affected by changes in DNA methylation in *IDH1/2* mutant cancers. These chromosomal regions included approximately corresponded to chromosomal bands 2p12, 4q28.3, 5p14.1, 9p21.3, 9p21.1, 10q21.1, 10q21.3, 10q23.1, and 11p12, which are dark bands in a standard G-banded 850 bands per haploid set (bphs) karyogram and historically labeled as heterochromatin (Fig. 3d). Notably, chromosome 13, which showed the highest fraction of hypermethylated probes, also showed a striking absence of common hypermethylated probes for gene bodies, enhancers and promoters in 13q31.1 and 13q31.3 further highlighting the propensity of *IDH1/2* induced hypermethylation only to particular parts of the chromosome. While these areas show good coverage by probes on Illumina arrays, there is high variability in the number of probes annotated as enhancers, promoters and gene bodies present in each of these regions (Supp. Fig. 3). This suggests that some areas of the chromosomes may not be the target of DNA methylation changes induced by *IDH1/2* mutation in cancers and may be regulated by other epigenetic mechanisms.

### Common pathway enrichment in *IDH 1/2* mutant tumors

**Figure 5:**
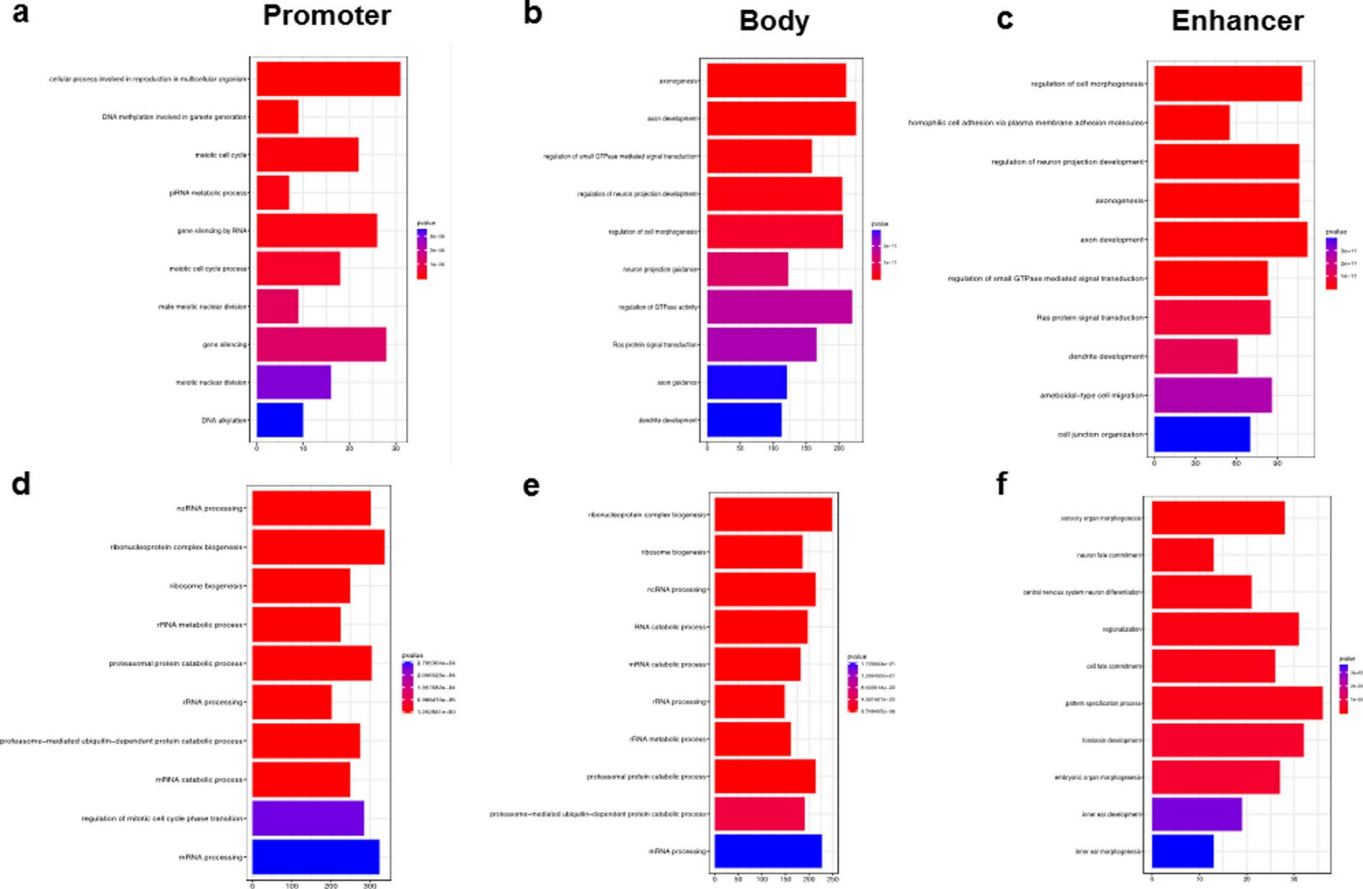
The effect of IDH1/2 mutation induced DNA methylation of cell biology. (**a**) Hypermethylated promoters show enrichment in GO terms for in meiosis and gamete generation, which likely have no role in cancer growth. (**b**) Gene bodies and (**c**) enhancers was enriched for GO terms associated with axonogenesis, dendrite development and neuronal differentiation, Ras signaling, cell morphogenesis GTPase activity as well as cell migration. (**d**) Hypomethylated promoter probes were associated with basic cellular functions such as RNA processing and cell proliferation. (**e**) Common hypomethylated probes in gene bodies also showed enrichment in GO terms for such as RNA processing and protein catabolism. (**f**) Hypomethylated enhancers were enriched for GO terms involved in cell fate commitment, early organ development, pattern specification and morphogenesis suggesting hypomethylation of tissue specific enhancers plays a in blockade of differentiation observed in *IDH1/2* mutant cancers.

Mutations act in concert when dysregulating cellular signaling in cancer. DNA methylation has a different functional effect on gene bodies, enhancers and promoters and a single mutation event on signaling is difficult to determine in archival human cancer samples. To investigate what cellular functions may be affected across all six *IDH1/2* mutant tumor types, we separately analyzed the effect of hyper- and hypomethylation on promoters, enhancers and gene bodies. Commonly hypomethylated promoters across all cancers showed enrichment of gene ontology (GO) terms associated with basic cellular functions such as RNA processing, regulation of mitosis, and ribosome biogenesis suggesting that genes likely critical in cellular functions are not silenced by global DNA hypermethylation. In contrary, GO associated with hypermethylated promoters were enriched (6/10 top GO terms) for genes involved in meiosis and gamete generation, likely playing no role in cancer. Hypermethylation of gene bodies and enhancers was enriched for GO terms associated with central nervous system function, such as axonogenesis, dendrite development, and neuronal differentiation. This observation could be due to a somewhat larger number of *IDH* mutant brain tumors in our cohort skewing the analysis towards cellular processes critical for brain development. Other GO terms affected by gene body and enhancer hypermethylation included Ras signaling, cell morphogenesis, GTPase activity, and cell migration (Fig. 5). The KEGG pathway analysis of hypermethylated probes in gene bodies and enhancers showed enrichment for MAPK, Rap1, and Ras signaling pathways (Supp. Fig. 4). Similar to gene promoters, the GO terms enriched in common hypomethylated probes in gene bodies were also enriched for basic cellular functions such as RNA processing, as well as RNA and protein catabolism. However, the hypomethylated enhancers were enriched for GO terms involved in cell fate commitment and early organ development and morphogenesis (Fig. 5, Supp. Fig. 5). Hypomethylated promoters and bodies were enriched in cellular senescence, RNA transport, and the mRNA surveillance pathway (Supp. Fig. 4). This suggests that only promoters regulating noncritical functions are safe to be hypermethylated in cancer cells and the hypomethylation of enhancers involved in cell differentiation may be the cause of previously observed inability to differentiate observed in *IDH* mutant cancers.

## Discussion

While the biochemical effect of *IDH1/2* mutations is well known^18^, the effect on tumorigenesis as well as tumor growth, and tumor progression remains less understood^36,37^. Variable prognosis of *IDH1/2* mutant tumors and response to *IDH* targeted therapy among different tumor types highlights the need to identify common and disease specific effects of *IDH1/2* mutations. The role of *IDH1/2* mutations in cancer has been puzzling since their discovery. While mutations in other metabolic enzymes such as fumarate hydratase and succinate dehydrogenase identified in hereditary leiomyomatosis, renal cell carcinoma and paragangliomas result in loss of function^38^, *IDH1/2* mutations are virtually always heterozygous, suggesting a gain of function as a possible carcinogenic mechanism. A universal feature of *IDH1/2* mutant cancers is the CpG island methylator phenotype resulting in global DNA hypermethylation^3,19,35^, due to the overproduction of 2-HG regardless of tumor type. While CpG islands are typically associated with promoters, there is increasing evidence that non-promoter orphan CpG islands are associated with enhancer activity^39–41^. Our data suggest that CpG islands identified as hypermethylated in *IDH* mutant cancers are in fact CpG islands with enhancer activity rather than promoters.

*IDH1/2* mutation alters canonical metabolic pathways^37,42^ including glutamine catabolism^43,44^ and the TCA cycle^45^. Lastly, the impact of *IDH1/2* targeting drugs has been modest so far and has various effects depending on the tumor type suggesting there is a substantial heterogeneity among *IDH* mutant cancers^20,46^. Furthermore, specific tumor types show distinct predisposition to specific *IDH1* or *IDH2* mutations. While *IDH2* mutations have been observed predominantly in acute myeloid leukemia^3^ and later in cholangiocarcinoma^10^ and solid papillary carcinoma with reverse polarity, a rare morphologic subtype of breast cancer^16^, *IDH1* mutations are strongly associated with low grade gliomas^9,26^. The association with low grade gliomas such as astrocytoma and oligodendroglioma is so strong, that World Health Organization (WHO) requires *IDH* mutation status for accurate diagnosis and prognosis. We show that there are disease specific regions as well as common regions of DNA/chromosomes that are preferentially affected across all *IDH* mutant cancers. *IDH* mutations do not directly cause hypermethylation, but rather lead to inhibition of demethylases and may preserve previously established tissue/cell-specific differences in DNA methylation patterns occurring before the mutation event. Nevertheless, our results show that enhancers associated with tissue differentiation and cell fate remain unmethylated. It is possible that the three dimensional structure of chromatin renders some parts of the chromatin more susceptible than others to *IDH* mutation induced hypermethylation.

Interestingly, although methylation of promoters and gene bodies showed almost the opposite direction genome-wide, the common hypomethylated probes in gene promoters and bodies were both enriched for GO terms for genes involved in basic cellular functions such as RNA processing and RNA and protein catabolism, suggesting that other epigenetic mechanisms may play a role in regulating critical cell functions in *IDH* mutant cancers. Both gene body and enhancer hypermethylation pathway analysis showed enrichment in the MAPK pathway suggesting a common signaling pathway in these tumors. Interestingly, although enhancers showed global hypermethylation, the enrichment of GO terms for hypomethylated enhancers was for processes involved in cellular and tissue differentiation. This is concordant with previous observations that IDH mutations “lock” cells in undifferentiated states and prevent terminal differentiation^19,22,25^. Our data suggest that this process may be mediated via hypomethylation of tissue specific enhancers. Interestingly, we observed that specific parts of the genome, coinciding with dark bands on chromosomal banding, show lack of common hyper- or hypomethylated probes for gene bodies, enhancers and promoters. This suggests that these particular parts of the chromatin may not be affected by *IDH1/2* mutation induced methylation changes, and their epigenetic regulation may depend on mechanisms other than DNA methylation^47^. While we have observed a striking predisposition, as well as a resistance, of specific chromosomal arms and regions to hypermethylation in *IDH1/2* mutant cancers, the major limitation of our study is the two-dimensional nature of our data which prohibits us to examine whether the affected regions interact with each other on a three-dimensional level. Our study identifies common effects of *IDH1/2* mutations on DNA methylation across six cancer types, derived from all three embryological layers. However it is limited by the fact that only in three cancers, AML, astrocytomas and oligodendroglioma we were able to perform a comparison with biologically relevant wild-type tumors and only in AML we compared the *IDH1/2* mutated tumors with the normal tissue.

We and others have previously shown that *IDH* mutation in gliomas leads to loss of insulation between topological domains and aberrant gene activation^25,48^. Whether there are also three-dimensional chromatin interactions that are common across all *IDH1/2* mutant cancers remains to be explored. While it is well known that oncometabolite 2-HG leads to global DNA hypermethylation, it is currently not clear whether hypermethylation affects DNA indiscriminately or whether functional or topographic parts of the genome are differentially methylated. Here we show that tumors with *IDH1/2* mutations can still be distinguished based on the tissue of origin suggesting that *IDH1/2* mutation induced methylation acts on the background of developmental epigenetic changes. In addition to previously described DNA hypermethylation phenotype, *IDH1/2* mutant tumors show surprisingly high level of DNA hypomethylation, which is particularly prominent across gene promoters. In striking contrast, DNA hypermethylation predominantly affects gene body regions and enhancers across all cancers; with exception of enhancers involved in terminal cell and tissue differentiation, which remain unmethylated. This suggests that the CpG hypermethylator phenotype induced by *IDH1/2* mutations affects predominantly non-promoter CpG islands with enhancer activity. Lastly, DNA methylation varies between chromosomes and chromosomal regions, which appear to be preferentially affected while other regions remain protected from the hypermethylation in *IDH* mutant setting. In summary, our findings provide new insight into topographic and functional effects of *IDH1/2* induced DNA methylation.

## Methods

### Patient samples and data generation

Raw methylation data from IDH-mutated acute myeloid leukemia (AML, N = 21) and cholangiocarcinoma (N = 9) were obtained from TCGA-LAML^6^ and TCGA-CHOL^11^ projects on The Cancer Genome Atlas (TCGA). See also Supplemental File 1. DNA methylation data from IDH mutated breast carcinoma (N = 5) with altered nuclear polarity and sinonasal undifferentiated carcinomas (N = 8) were obtained from our previously published cohorts^12,16^. In addition, DNA was extracted from the de-identified tumors of 20 patients with IDH mutated oligodendroglioma and 31 patients with IDH mutated astrocytoma who had been treated at the New York University Langone Medical Center (NYULMC). DNA was extracted from archival formalin-fixed paraffin-embedded (FFPE) tissue from tumor biopsies using automated Maxwell Promega system, and methylation profiling was performed at the NYU Department of Molecular Pathology using the Illumina Infinium Human Methylation 450 Bead-Chip (450 K array) or Illumina EPIC array according to the manufacturer’s instructions as described previously^49^. All NYU brain tumor samples had *IDH1/2* status identified by clinically validated CLIA certified NYU NGS50 IonTorrent sequencing panel and had the methylation subgroup confirmed using CNS tumor classifier tool as described previously^33^. All DNA methylation profiles across cancer types were analyzed together regardless of the mutated *IDH1* or *IDH2 gene*, or mutation hotspot. The research was performed in accordance with institutional and federal guidelines and regulations pertaining to the discarded tissue research. The study was approved by the NYU Institutional Review Board and the waiver of consent was granted (IRB approval#: NYU# S14-00948).

### Methylation data analysis of IDH mutated tumors

Raw methylation data were then analyzed with the R Bioconductor package *Minfi*, which was used to subset for overlapping probes among the two different methylation array platforms via the *CombineArrays* function. *Minfi* was used to check for the quality of samples by calculating the mean detection p-values. Samples with mean p < 0.01 were considered for further downstream analysis. Sex probes were removed, and all samples passed quality control. All sample probes were quantile normalized, adjusted for background signal, and processed for identifying the differentially methylated CpG sites. Stratified quantile normalization (*preprocessQuantile*) was chosen to control for differences between type I and type II probes. The number of overlapping probes between the two arrays after quality control and filtering was 363,700.

M-values, which are calculated using the log2 ratio of the intensities of methylated probes versus unmethylated probes, were used as they have been suggested to be more statistically valid than beta values for differential methylation analysis. The differential methylation analysis sorted the probes in order of smallest to largest q-values, so that the top probes represent the sources of largest variation between sample groups. Heatmaps were generated from the top 10,000 probes from this list using hierarchical clustering via *Minfi* to display global methylation pattern differences and similarities between samples. Average M-values were calculated in R for each probe across each tumor type. For each tumor type, hypomethylated probes were then extracted from the full list of overlapping probes by subsetting for a mean M-value cutoff of −2, and hypermethylated probes were extracted by subsetting for a mean M-value cutoff of +2. These values correspond to beta values of 0.2 and 0.8, respectively^50^. Mean global methylation levels were determined for each tumor type. Hypermethylated and hypomethylated probe lists for each tumor type were further subsetted into enhancer regions, regions within 200 base pairs of a transcriptional start site (TSS, or promoters), and gene body regions. This was done using the gene region information provided by the University of California Santa-Cruz for each probe on the reference annotation files of the Illumina arrays. If probes were associated with more than one gene region, they were included in the lists for each region.

### Determining common and tumor type specific hyper-/hypomethylated probes and chromosome specific DNA methylation analysis

The *InteractiVenn* online tool was used to generate Venn diagrams that display both the overlapping and unique hypermethylated or hypomethylated probes between the six tumor types, using default settings. R was used find the intersections between the lists of hypermethylated probes for each tumor type and between the lists of hypomethylated probes for each tumor type. This resulted in lists of probes either commonly hypomethylated or commonly hypermethylated across tumor types. The *setdiff* function then allowed for identification of probes that were uniquely hypomethylated or uniquely hypermethylated for each tumor type.

Using these lists, the karyoploteR R package was used to plot the distribution of hypermethylated and hypomethylated probes for each tumor type and gene region across the entire genome, versus mean methylation in M-values. These probes therefore all already met the criteria of a minimum cutoff M-value of +2 for hypermethylation, and a maximum M-value cutoff of −2 for hypomethylation. The karyoploteR package was also used to examine probe coverage across the genome as a control to identify any regions that did not have any probes to begin with. This was done by plotting all overlapping probes between 450 K and 850 K arrays based on their position alone (Supp. Fig. 4). A similar approach was used when plotting all common hypermethylated and hypomethylated probes with gene body, promoter, and enhancer probes combined (Supp. Fig. 4). By comparing mean methylation levels across all probes, we were able to identify the regions of the genome that are consistently hypermethylated and hypomethylated for each tumor type.

The total number of hypomethylated and hypermethylated probes for each chromosome arm, gene region, and tumor type was extracted using R, based on centromere position information obtained from the UCSC Table Browser retrieval tool. The human genome assembly and database used were GRCh37/hg19 and hg19, respectively. The *gap* table was then filtered for centromeres only. Acrocentric chromosome arms, namely 13p, 14p, 15p, 21p, and 22p, were excluded from data analysis, as they contained no hypermethylated or hypomethylated probes. Ratios were created and plotted between the number of probes associated with a particular gene region per arm for each tumor type and methylation state and the total number of probes associated with a particular gene region per arm. Similar analyses were performed for common hypermethylated and hypomethylated probes as well. Ratios were also determined and plotted between common hypermethylated or hypomethylated probes and all overlapping probes between array types. Two-tailed two sample t-tests assuming unequal variances were performed between the ratios of the p and q arms for each chromosome and the ratios for the p and q arms across the entire genome.

### Analyzing differential methylation of IDH mutated and IDH wild-type tumors

IDH mutated astrocytomas were compared with IDH wild-type glioblastomas (GBM, n = 20), which included samples from a Classic subtype (RTKII, n = 10) and Mesenchymal subtype (n = 10). IDH mutated oligodendrogliomas were compared with Proneural (RTKI) GBMs (n = 10). IDH mutated cholangiocarcinomas were compared with wild-type cholangiocarcinomas (n = 7) and IDH mutated AML samples were compared with IDH wild-type AML (n = 100) and a cohort of normal non-neoplastic blood samples of patients evaluated for leukocytosis (n = 32). Raw methylation data for IDH wild-type cholangiocarcinoma and AML samples were downloaded from TCGA, and methylation data for IDH wild-type GBM samples were obtained from our previously published cohorts that were processed on the Illumina Infinium Human Methylation 450 Bead-Chip (450 K array) or Illumina EPIC array^33^. IDH mutation statuses for NYU tumors were clinically validated and confirmed using the online DNA methylation classifier, and mutation statuses for TCGA tumors were obtained from the project supplemental file.

Differential methylation was performed using Minfi for each of the pairs using the same settings described previously, and selecting only probes with an FDR < 0.05. The total number of differentially expressed genes with an FDR < 0.05 varied as follows: 271,300 (IDH Oligodendroglioma vs GBM RTKI), 243,435 (IDH Astrocytoma vs GBM RTKII/GBM Mesenchymal), 980 (IDH vs wild-type Cholangiocarcinoma), and 254,537 (IDH vs wild-type AML).Due to the low number of probes that passed FDR < 0.05, cholangiocarcinoma comparison was excluded from further analysis. Heatmaps were generated via hierarchical clustering for each pair using only the top 10,000 most differentially methylated probes. Average M-values of −2 and +2 across all samples within a given tumor type were used as cutoffs for extracting hypomethylated and hypermethylated probes, respectively, from each list of significant differentially methylated probes. Identified tumor specific hyper- and hypomethylated probes for IDH mutated tumors were then separated into gene body regions, TSS regions, and enhancer regions using the UCSC gene region information provided for each probe by Illumina. KaryoploteR was used to plot the unique hypermethylated and hypomethylated probe distributions between each pair for each gene region, across all autosomal chromosomes. The y-axis scale (representing mean M-values) for each pair had a range of −/+1.9 to −/+5. Bar plots were plotted using the number of probes in different regions and two-tailed t-tests assuming unequal variances were performed to obtain p-value for each pair comparison.

### Pathway and gene ontology analysis

Common hypermethylated and hypomethylated probes across all the disease types were used to determine the GO terms and pathways they were involved in. Common hypermethylated and hypomethylated probes were categorized as promoters, enhancers and gene bodies based on the Illumina annotation. Each set of probes were run through the Gene Ontology enrichment analysis using R package *ClusterProfiler*. The bar plots represent the number of genes (x-axis) involved in specific GO terms (y-axis) based on the biological processes. The color of the bar shows the significance level of each term. *Cytoscape* was used to generate the network plots using the results of GO enrichment. Network plots show the top 10 genes in each GO term that are interconnected by certain common genes. The size of the node represents the number of genes involved in each GO category (as represented in bar plot). KEGG enrichment from *ClusterProfiler* was used to find the signaling pathways based on each set of probes. The dot plots represent ratio of genes (x-axis) involved in each signaling pathway (y-axis) of KEGG database. Size of the dots shows the gene counts and the color denotes the significance level.

## Supplementary information

Supplementary information 

## Data Availability

NYU derived DNA methylation data can be accessed at the NCBI GEO repository (accession #GSE124617). Analysis R code can be accessed at https://github.com/rbledea/Functional-and-Topographic-Effects-on-DNA-Methylation-Across-IDH1-2-Mutant-Cancers.

## References

[1] Dang L (2009). Cancer-associated IDH1 mutations produce 2-hydroxyglutarate. Nature.

[2] Yen KE, Bittinger MA, Su SM, Fantin VR (2010). Cancer-associated IDH mutations: biomarker and therapeutic opportunities. Oncogene.

[3] Figueroa ME (2010). Leukemic IDH1 and IDH2 mutations result in a hypermethylation phenotype, disrupt TET2 function, and impair hematopoietic differentiation. Cancer Cell.

[4] Kosmider O (2010). Mutations of IDH1 and IDH2 genes in early and accelerated phases of myelodysplastic syndromes and MDS/myeloproliferative neoplasms. Leukemia.

[5] Welch JS (2012). The origin and evolution of mutations in acute myeloid leukemia. Cell.

[6] Cancer Genome Atlas Research N (2013). Genomic and epigenomic landscapes of adult de novo acute myeloid leukemia. N Engl J Med.

[7] Yan H (2009). IDH1 and IDH2 mutations in gliomas. N Engl J Med.

[8] Watanabe T, Nobusawa S, Kleihues P, Ohgaki H (2009). IDH1 mutations are early events in the development of astrocytomas and oligodendrogliomas. Am J Pathol.

[9] Juratli TA (2012). IDH mutations as an early and consistent marker in low-grade astrocytomas WHO grade II and their consecutive secondary high-grade gliomas. J Neurooncol.

[10] Borger DR (2012). Frequent mutation of isocitrate dehydrogenase (IDH)1 and IDH2 in cholangiocarcinoma identified through broad-based tumor genotyping. Oncologist.

[11] Farshidfar F (2017). Integrative Genomic Analysis of Cholangiocarcinoma Identifies Distinct IDH-Mutant Molecular Profiles. Cell Rep.

[12] Dogan S (2017). Frequent IDH2 R172 mutations in undifferentiated and poorly-differentiated sinonasal carcinomas. J Pathol.

[13] Jo VY, Chau NG, Hornick JL, Krane JF, Sholl LM (2017). Recurrent IDH2 R172X mutations in sinonasal undifferentiated carcinoma. Mod Pathol.

[14] Amary MF (2011). IDH1 and IDH2 mutations are frequent events in central chondrosarcoma and central and periosteal chondromas but not in other mesenchymal tumours. J Pathol.

[15] Amary MF (2011). Ollier disease and Maffucci syndrome are caused by somatic mosaic mutations of IDH1 and IDH2. Nat Genet.

[16] Chiang S (2016). IDH2 Mutations Define a Unique Subtype of Breast Cancer with Altered Nuclear Polarity. Cancer Res.

[17] Snuderl M (2015). Deep sequencing identifies IDH1 R132S mutation in adult medulloblastoma. J Clin Oncol.

[18] Losman JA, Kaelin WG (2013). What a difference a hydroxyl makes: mutant IDH, (R)-2-hydroxyglutarate, and cancer. Genes Dev.

[19] Lu C (2012). IDH mutation impairs histone demethylation and results in a block to cell differentiation. Nature.

[20] Rohle D (2013). An inhibitor of mutant IDH1 delays growth and promotes differentiation of glioma cells. Science.

[21] Wang F (2013). Targeted inhibition of mutant IDH2 in leukemia cells induces cellular differentiation. Science.

[22] Saha SK (2014). Mutant IDH inhibits HNF-4alpha to block hepatocyte differentiation and promote biliary cancer. Nature.

[23] Kats LM (2014). Proto-oncogenic role of mutant IDH2 in leukemia initiation and maintenance. Cell Stem Cell.

[24] Kannan K (2012). Whole-exome sequencing identifies ATRX mutation as a key molecular determinant in lower-grade glioma. Oncotarget.

[25] Modrek AS (2017). Low-Grade Astrocytoma Mutations in IDH1, P53, and ATRX Cooperate to Block Differentiation of Human Neural Stem Cells via Repression of SOX2. Cell Rep.

[26] Olar A (2015). IDH mutation status and role of WHO grade and mitotic index in overall survival in grade II-III diffuse gliomas. Acta Neuropathol.

[27] Thol F (2010). IDH1 mutations in patients with myelodysplastic syndromes are associated with an unfavorable prognosis. Haematologica.

[28] Tefferi A (2012). IDH mutations in primary myelofibrosis predict leukemic transformation and shortened survival: clinical evidence for leukemogenic collaboration with JAK2V617F. Leukemia.

[29] DiNardo CD (2015). Characteristics, clinical outcome, and prognostic significance of IDH mutations in AML. Am J Hematol.

[30] Patnaik MM (2012). Differential prognostic effect of IDH1 versus IDH2 mutations in myelodysplastic syndromes: a Mayo Clinic study of 277 patients. Leukemia.

[31] Goyal L (2015). Prognosis and Clinicopathologic Features of Patients With Advanced Stage Isocitrate Dehydrogenase (IDH) Mutant and IDH Wild-Type Intrahepatic Cholangiocarcinoma. Oncologist.

[32] Wang P (2013). Mutations in isocitrate dehydrogenase 1 and 2 occur frequently in intrahepatic cholangiocarcinomas and share hypermethylation targets with glioblastomas. Oncogene.

[33] Capper D (2018). DNA methylation-based classification of central nervous system tumours. Nature.

[34] Sturm D (2012). Hotspot mutations in H3F3A and IDH1 define distinct epigenetic and biological subgroups of glioblastoma. Cancer Cell.

[35] Turcan S (2012). IDH1 mutation is sufficient to establish the glioma hypermethylator phenotype. Nature.

[36] Laurence MG, Boulay K, Topisirovic I, Huot ME, Mallette FA (2017). Oncogenic Activities of IDH1/2 Mutations: From Epigenetics to Cellular Signaling. Trends Cell Biol.

[37] Tateishi K (2015). Extreme Vulnerability of IDH1 Mutant Cancers to NAD+ Depletion. Cancer Cell.

[38] Raimundo N, Baysal BE, Shadel GS (2011). Revisiting the TCA cycle: signaling to tumor formation. Trends Mol Med.

[39] Bae MG, Kim JY, Choi JK (2016). Frequent hypermethylation of orphan CpG islands with enhancer activity in cancer. BMC Med Genomics.

[40] Bell JSK, Vertino PM (2017). Orphan CpG islands define a novel class of highly active enhancers. Epigenetics.

[41] Deaton AM, Bird A (2011). CpG islands and the regulation of transcription. Genes Dev.

[42] Reitman ZJ (2014). Cancer-associated isocitrate dehydrogenase 1 (IDH1) R132H mutation and d-2-hydroxyglutarate stimulate glutamine metabolism under hypoxia. J Biol Chem.

[43] Seltzer MJ (2010). Inhibition of glutaminase preferentially slows growth of glioma cells with mutant IDH1. Cancer Res.

[44] Metallo CM (2012). Reductive glutamine metabolism by IDH1 mediates lipogenesis under hypoxia. Nature.

[45] Grassian AR (2014). IDH1 mutations alter citric acid cycle metabolism and increase dependence on oxidative mitochondrial metabolism. Cancer Res.

[46] Nassereddine S, Lap CJ, Haroun F, Tabbara I (2017). The role of mutant IDH1 and IDH2 inhibitors in the treatment of acute myeloid leukemia. Ann Hematol.

[47] Turcan S (2018). Mutant-IDH1-dependent chromatin state reprogramming, reversibility, and persistence. Nat Genet.

[48] Flavahan WA (2016). Insulator dysfunction and oncogene activation in IDH mutant gliomas. Nature.

[49] Serrano J, Snuderl M (2018). Whole Genome DNA Methylation Analysis of Human Glioblastoma Using Illumina BeadArrays. Methods Mol Biol.

[50] Du P (2010). Comparison of Beta-value and M-value methods for quantifying methylation levels by microarray analysis. BMC Bioinformatics.

