# Supplementary material for: Functional and topographic effects on DNA methylation in IDH1/2 mutant cancers
Source: Sci Rep. 2019 Nov 14;9:16830. doi: 10.1038/s41598-019-53262-7 (PMC6856069; doi:10.1038/s41598-019-53262-7)
Supplement: Supplementary file 1 — Supplementary information [file 41598_2019_53262_MOESM1_ESM.pdf]

Functional and topographic effects on DNA methylation in *IDH1/2* mutant cancers

Ramona Bledea[1], Varshini Vasudevaraja[1], Seema Patel[1], James Stafford[2], Jonathan Serrano[1], Gianna Esposito[1], Lilian M Tredwin[1], Nina Goodman[1], Andreas Kloetgen[1], John G. Golfinos[3], David Zagzag[1, 3], Britta Weigelt[4], A. John Iafrate[5], Erik P. Sulman[6], Andrew S. Chi[7], Snjezana Dogan[4], Jorge S. Reis-Filho[4], Sarah Chiang[4], Dimitris Placantonakis[3, 7, 8, 9], Aristotelis Tsirigos[1, 7], Matija Snuderl[1, 7*]

[1] Department of Pathology, NYU Langone Health and School of Medicine, New York, NY, USA.

[2] Department of Neurological Sciences, University of Vermont, Larner College of Medicine, Burlington, VT, USA

[3] Department of Neurosurgery, NYU Langone Health and School of Medicine, New York, NY, USA.

[4] Department of Pathology, Memorial Sloan Kettering Cancer Center, New York, NY, USA.

[5] Department of Pathology, Massachusetts General Hospital, Boston, MA, USA

[6] Department of Radiation Oncology, NYU Langone Health and School of Medicine, New York, NY, USA.

[7] Laura and Isaac Perlmutter Cancer Center, NYU Langone Health and School of Medicine, New York, NY, USA.

[8] Kimmel Center for Stem Cell Biology, NYU Langone Health and School of Medicine

[9] Neuroscience Institute, NYU Langone Health and School of Medicine

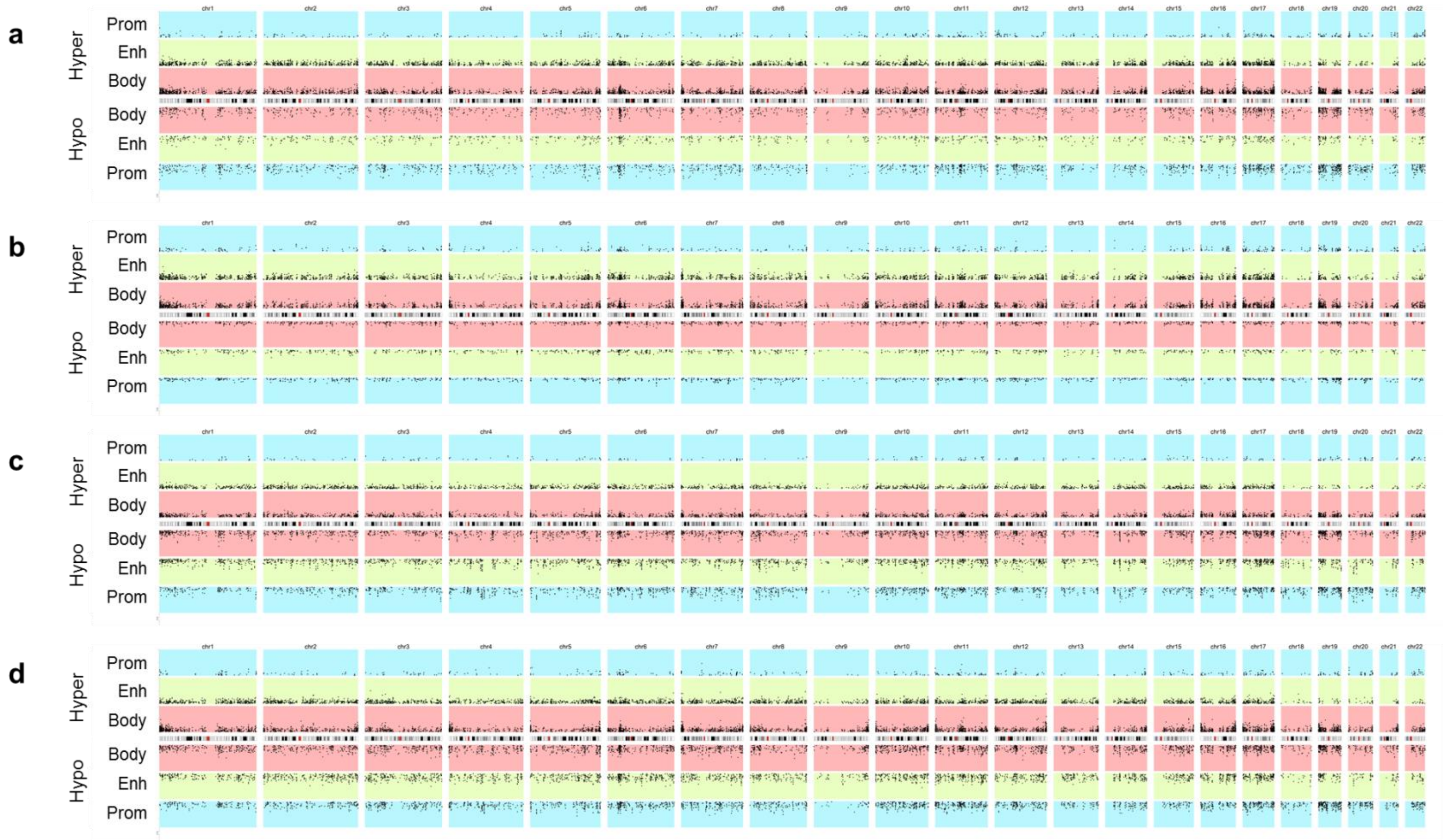

Supplemental Figure 1.

Karyoplots plotting the tumor specific hypermethylated and hypomethylated probes identified from comparisons with *IDH1/2* wild-type samples between each pair for each functional region, across all autosomal chromosomes. For hypermethylated probes, the y-axis values range from +1.9 to +5 and for hypomethylated probes the range is between -1.9 and -5. Distribution of disease specific hyper- hypomethylated sites in IDH mutated AML in comparison with normal blood (**a**), or IDH wild-type AML (**b**), IDH mutated astrocytoma compared to IDH wild-type GBM (Classic and Mesenchymal) (**c**) and IDH mutated oligodendroglioma compared to Proneural (RTKI GBM) (**d**) shows gene bodies and enhancers with a higher number of hypermethylated probes across chromosomes and low number of hypermethylated promoter probes in all tumor types. In contrast, the number of hypomethylated promoters is high across all chromosomes and all tumor types. See also Figure 1 for quantification.

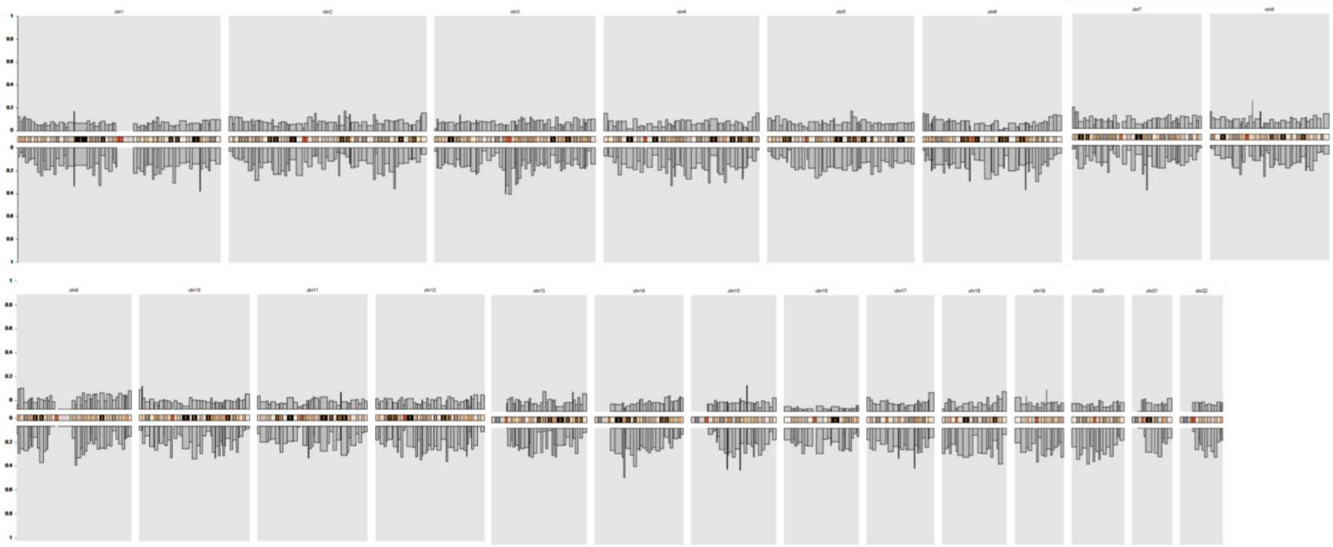

Supplemental Figure 2.

Distribution of common hyper and hypomethylated probes across the genome and ratio of common hyper- and hypomethylated probes per each chromosomal band. In addition to expected hypermethylation, we also observed a profound hypomethylation across the genome.

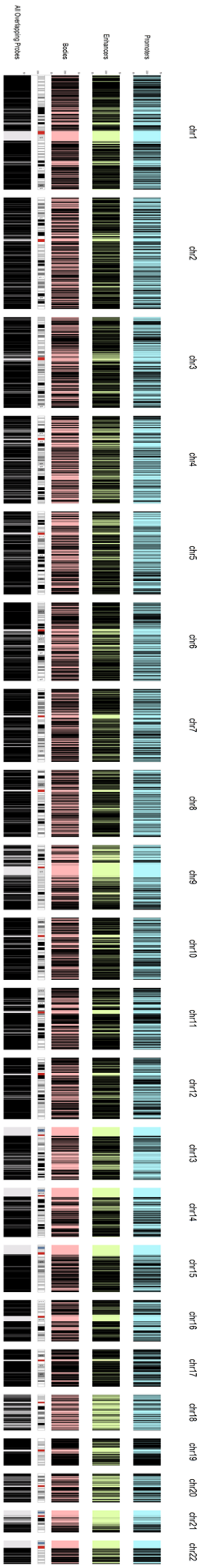

Supplemental Figure 3.

Comparison of the 450k and EPIC arrays coverage. The probe chromosomal distribution of all overlapping probes, as well as gene body, promoter and enhancer annotated probes on Illumina 450k and Illumina EPIC arrays.

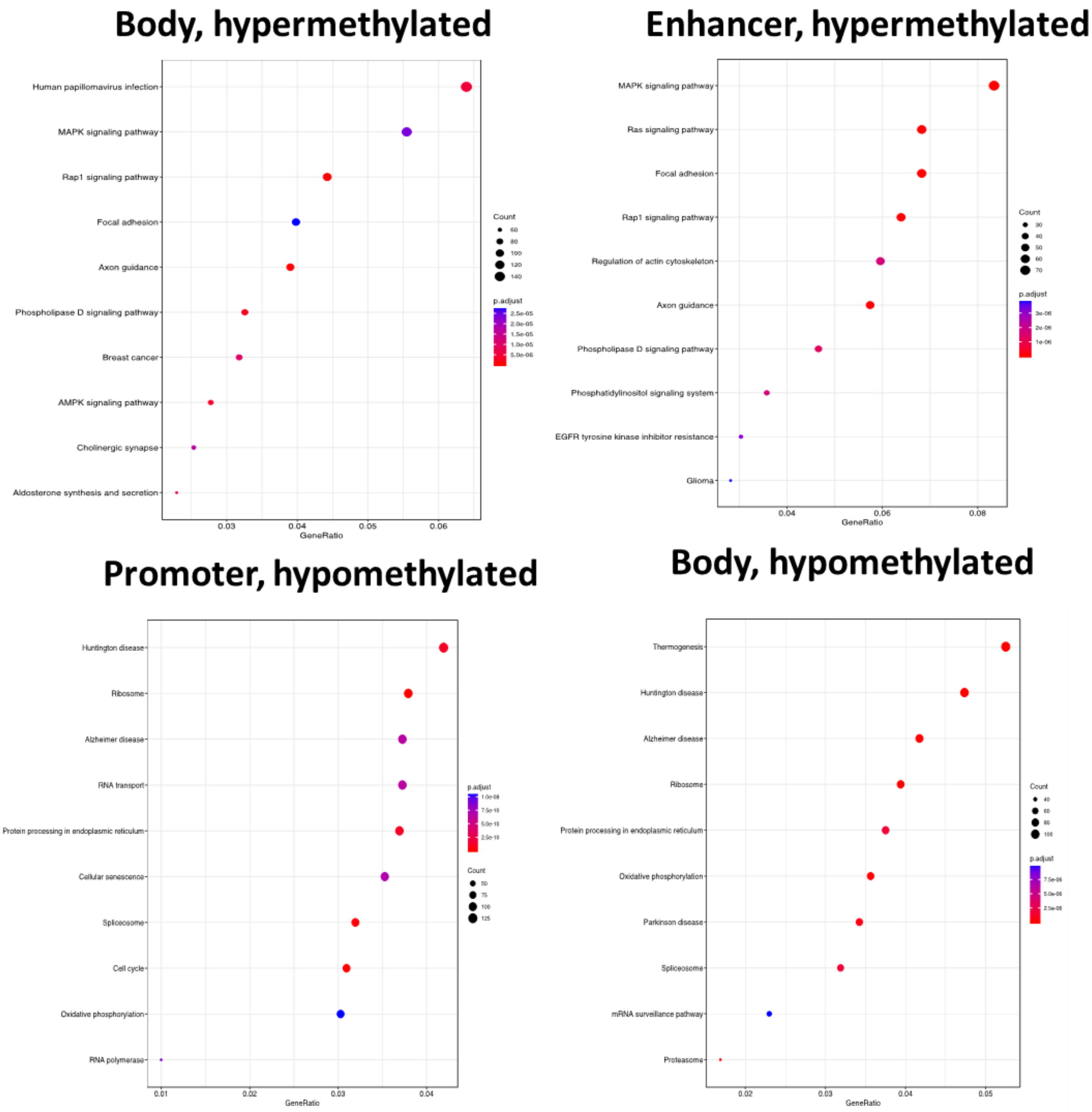

Supplemental Figure 4.

The KEGG pathway analysis. Sufficient probes were available to analyze pathways affected by hypermethylated gene body probes and enhancer probes and common hypomethylated promoter and gene body probes. The pathway analysis of hypermethylated probes in gene bodies and enhancers showed enrichment for MAPK, Rap1 and Ras signaling pathways while hypomethylated promoters show enrichment in cellular transport, senescence and neurodegenerative diseases.

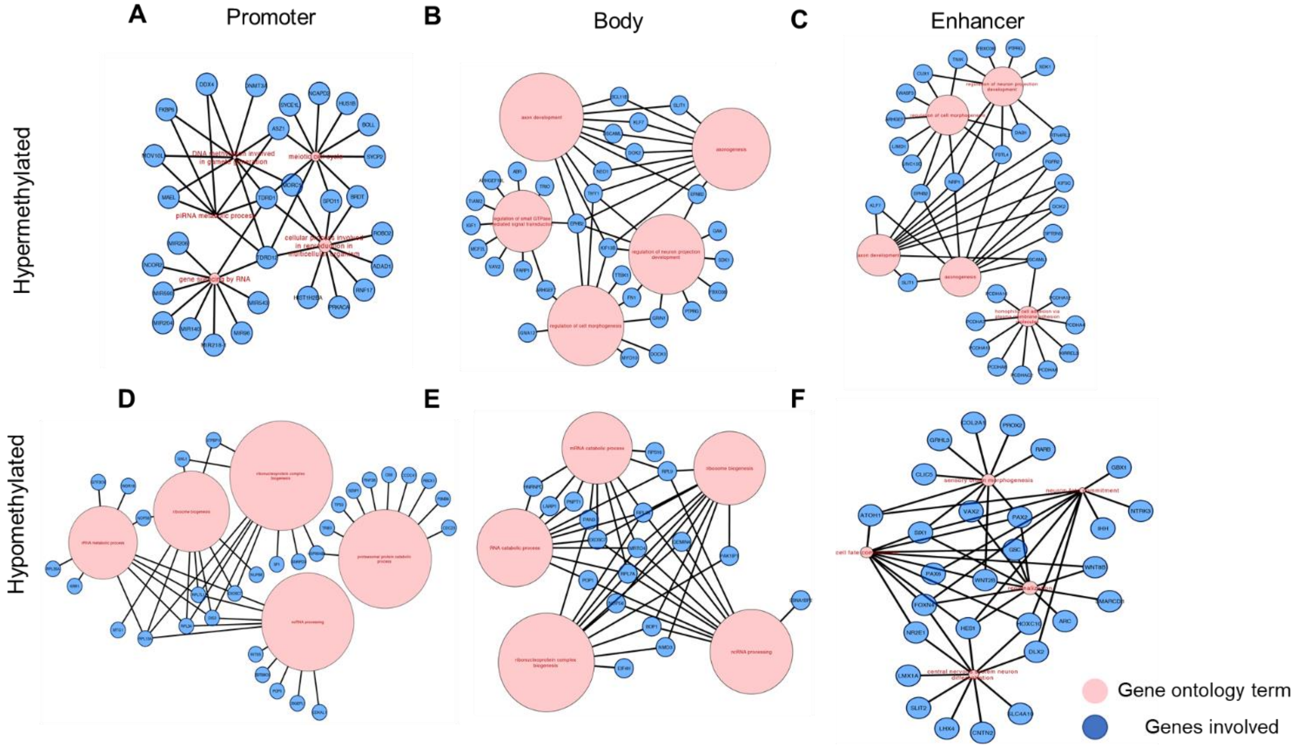

Supplemental Figure 5

Genes associated with enriched GO terms. GO terms with top 10 associated genes are shown for hypermethylated promoter (**A**), gene body (**B**) and enhancers (**C**), and hypomethylated promoter (**D**), gene body, (**E**) and enhancer (**F**).
